# Optical vortex beam controlling based on fork grating stored in a dye-doped liquid crystal cell

**DOI:** 10.1038/s41598-022-25779-x

**Published:** 2022-12-08

**Authors:** P. Soleimani, H. Khoshsima, M. Yeganeh

**Affiliations:** Faculty of Physics, University of Tabriz, Tabriz, Iran

**Keywords:** Optics and photonics, Applied optics, Optical materials and structures, Optical physics, Optical techniques, Other photonics

## Abstract

In this paper, we investigate the generation and controlling of the optical vortex beam using a dye-doped liquid crystal (DDLC) cell. The spatial distribution of the quasi-sinusoidal orientation of the liquid crystal molecules creates a quasi-sinusoidal phase grating (PG) in the DDLC cell. Depending on the incident light pattern, Trans to Cis photoisomerization of the dye molecules affects the orientation of the liquid crystal molecules. To do so, an amplitude fork grating (FG) is used as a mask, and its pattern is stored in the cell by a pattern printing method as the PG. One of the particular features of the stored grating in the cell is its capability in the diffraction efficiency controlled by the applied electric field. The results show, based on the central defect in the FG pattern, the diffracted probe beam in different orders is optical vortices. As a new technique, this type of stored pattern acts like an amplitude grating but according to the results, its structure is in fact a PG. This technique leads to the vortex beam switching capability by applying an electric field to the cell. The results show that by applying 22 V, all the diffraction orders vanish. Meanwhile, the vortex beams reappear by removing the applied voltage. The diffraction efficiency of the vortex beams as well as its generation dependency on the polarization of the incident beam studied. The maximum efficiency of the first diffraction order for linear polarized incident beam was obtained at 0 V, about 8%. Based on the presented theory, a simulation has been done which shows the Cis form of the dye molecules has been able to change the angle of LC molecules on average about 12.7°. The study of diffracted beam profiles proves that they are electrically controllable vortex beams.

## Introduction

The development of beam shaper devices has enhanced the ability to manipulate laser beam profiles over the last three decades^1–3^. The attractive features of optical vortices, both in terms of theoretical considerations and their technical applications, have appealed to the attention of scientists both in the science and technology fields^4^. Profile shaping of laser beams is done by two methods, that is, amplitude and phase modulation of laser beams^5,6^. Phase modulation techniques generate vortex beams without wasting energy, so these methods are highly efficient in practice^3^. Despite amplitude modulation methods being effective, they waste a lot of laser energy. In general, there are various ways to generate vortex beams including spatial light modulators (SLM)^7^, spiral phase plates^8^, Q-plates^9,10^, and also amplitude gratings which have a singularity, similar to the fork gratings^11^. A novel optical device is introduced in this experimental project, based on beam phase modulation using a liquid crystal (LC) cell. Its structure as a q-plate contains a singularity in the transverse refractive index distribution with a topological charge printed on it. This device is able to convert a Gaussian beam into a vortex beam with orbital angular momentum (OAM) which has a wide variety of applications in many fields of optics, including optical tweezers^12,13^, imaging^14^, and quantum information^15^. Although obviously one of the simple methods for generating the vortex beam is utilizing FGs^16^, sure LC cells are the best choice for generating controllable vortex beams^17^. The advantages of this kind of device are the simplicity of its construction and ease of use and control in comparison with SLM which needs graphic processors and a complex electronic control system and also has disadvantages due to the diffraction effects of its pixelated structure.

Road like LC molecules usually being anisotropic and combined with their associated behavior in the bulk form leads to these systems exhibiting unique optical properties. However, their optical behavior significantly changes when a small amount of guest substance affects the properties of a host material^18,19^. So, the Guest–Host orientational interaction in liquid crystalline materials, by doping LC with azo dye, affects the optical properties of the LC molecules^20,21^. By controlling the transverse distribution of this kind of orientational interaction, a phase grating across the LC cell with a singularity in the center of the sample is created^22^. In this article, a simple method is suggested for the formation of quasi-sinusoidal phase grating that could be used to control the generation of vortex optical beam in a homogeneously doped E7 liquid crystal with 1 wt% methyl red (MR) dye.

## Theory

Gratings are a simple and easy technique to generate arbitrary wavefronts^23^. In early 1990, the usage of diffractive optical elements for converting spatial coherence beams to optical vortex beams was also investigated^24^. Bazhenov, and et al. showed that when the diffraction grating contains edge nonlocality in the center (like a fork-grating), an optical singularity is observed in diffracted beams which are determined by l which corresponds to the difference between the number of upper and lower lines. Depending on their preparation methods, fork grating can be a linear structure either in amplitude or phase which form a grating in which the difference in fringe intervals in the upper and lower halves forms a kind of singularity that can produce different vortex beams according to the size of the topological vortex charge^25–27^. Vortex topological charge is not affected by changes in recording conditions, such as amplitude and phase modulation^28^. In general, the transmittance function of a sinusoidal amplitude Fork grating can be written as follows:

1 $${t}_{\mathrm{SA}}\left(x ,y\right)=A+B\mathrm{cos}\left(Gx-p\theta \right)$$

in which A and B are two constants, $\theta$ is the azimuthal angle in the polar coordinates and $G=\frac{2\uppi }{d}$, d is the spatial period of the grating far from the singularity point and p is an integer. For such gratings with *p*_=_1, only three diffraction orders, (− 1, 0, and + 1) can be produced. So, for a fork grating with a higher *p* integer, only − *p*, 0 and + *p* charged vortices can be produced. If a binary amplitude grating is used instead of sinusoidal amplitude fork grating, multiple orders can be realized and in this case its transmittance function is given by:

2 $${t}_{\mathrm{BA}}\left(x ,y\right)=A+B\mathrm{sgn}\left[\mathrm{cos}\left(Gx-p\theta \right)\right]$$

**Figure 1:**
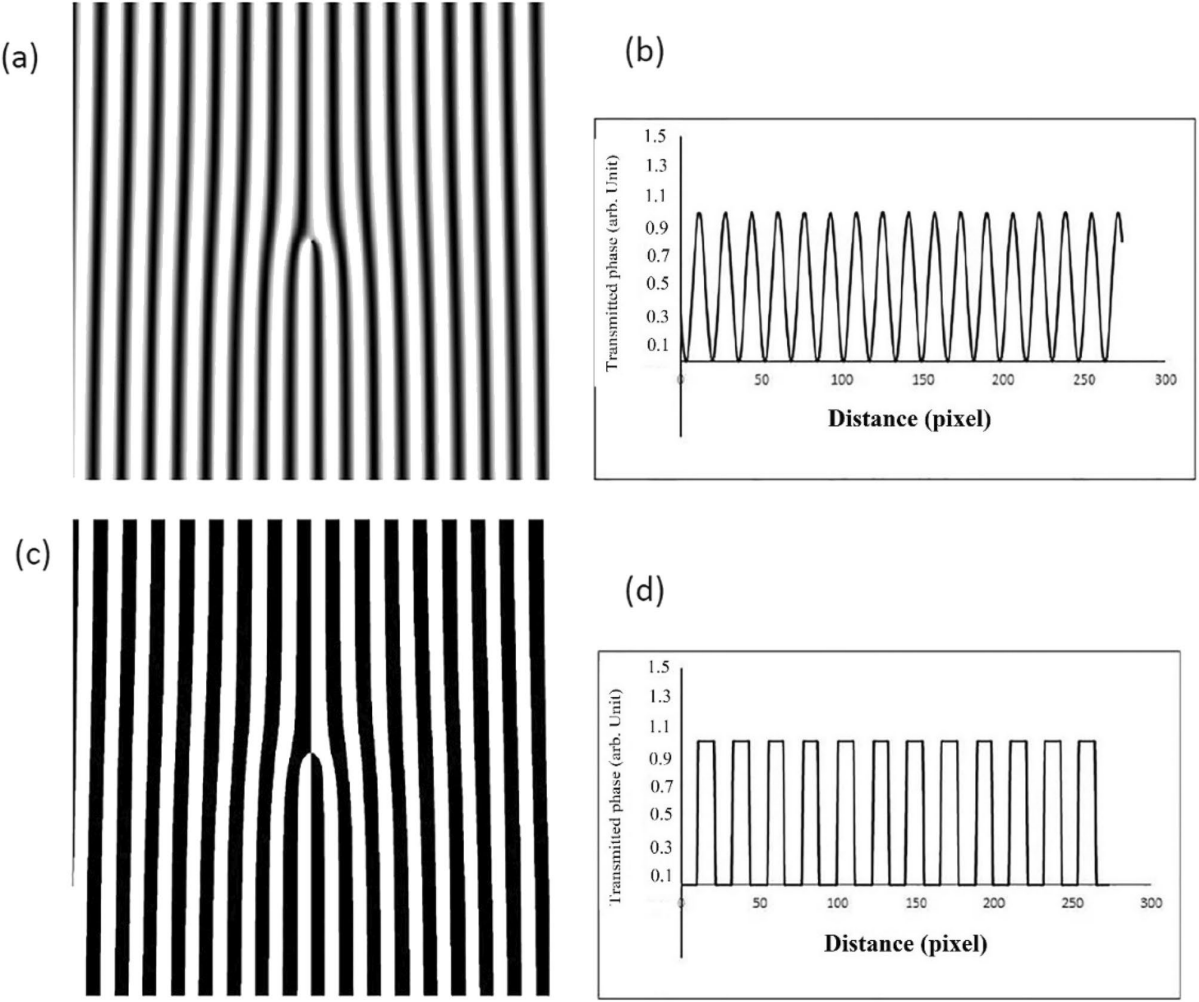
(**a**) Sinusoidal phase fork grating, (**b**) transmitted phase profile of sinusoidal phase fork grating, (**c**) binary phase fork grating, (**d**) transmitted phase profile of binary fork grating.

The plot of the transmittance function of sinusoidal/binary amplitude fork gratings and their transmittance profile is shown in Fig. 1^28^.

As an alternative to amplitude gratings, it is possible to use phase gratings. In this case, the phase of transmitted light modulates by the grating instead of its amplitude. For Liquid Crystal based phase gratings, the refractive index anisotropy ($\Delta n={n}_{e}(\theta )-{n}_{o}$) depends on the applied voltage because of the voltage dependency of the LC director ($\widehat{n}$) reorientation angle. Without any applied voltage, the director is parallel to the cell microgrooves ($\widehat{n} \parallel \overrightarrow{L}$), and the refractive index of the LC cell for incident light polarized parallel to the $\overrightarrow{L}$ will be ${n}_{e}$. By increasing the applied voltage, the director will reorient, and the refractive index decreases and reaches to the ${n}_{e}\left(\theta \right)$ which is closer to ordinary refractive index ${n}_{o}$, and at higher voltages leads to vanishing the refractive index anisotropy.

The spatially modulated refractive index of this kind of gratings in a given voltage:

3 $${n}_{g}\left(x,y\right)={n}_{m}+\frac{1}{2}\Delta n\left[\mathrm{cos}\left(Gx-p\theta \right)\right]$$

**Figure 2:**
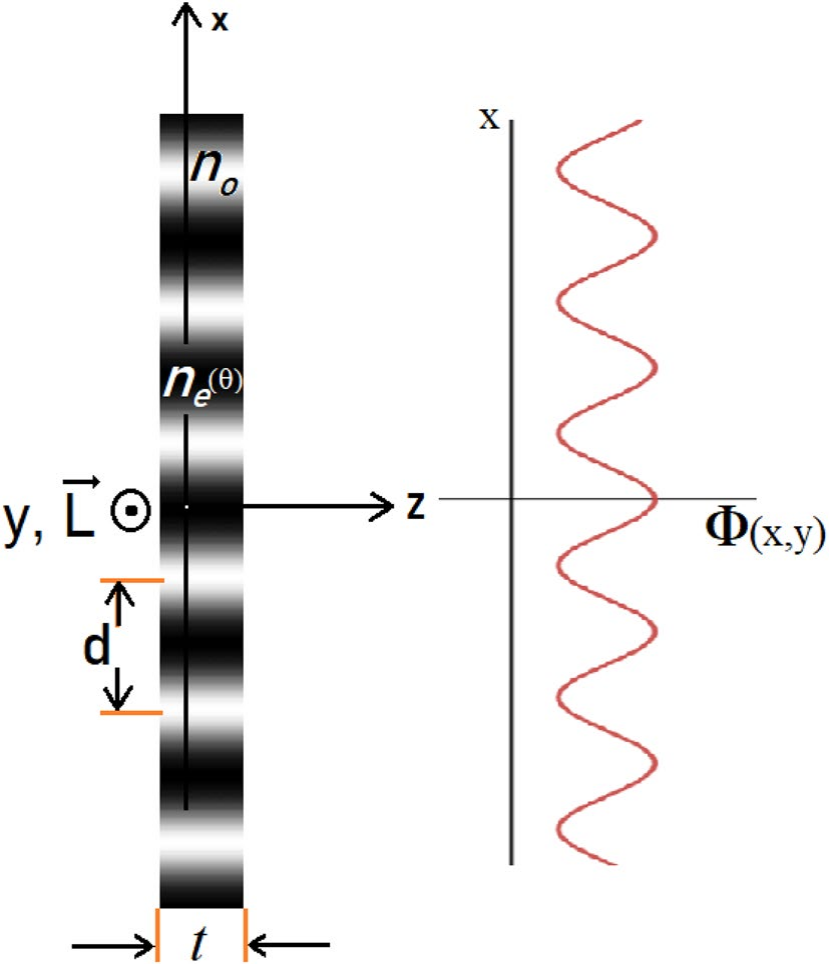
Sinusoidal phase grating inside the LC cell.

In which, ${n}_{m}=\frac{{n}_{e}(\theta )+{n}_{o}}{2}$ is the mean refractive index. We consider that the light wave propagates into the LC cell at normal incidence and as it will be shown in the results the absorption loss of the medium can be ignored. As it is shown in Fig. 2, by dropping the constant phase of transmitted light related to the LC cell walls and also the initial phase of the incident monochromatic plane wave, the phase of the light at the exit plane of the cell is given by,

4 $$\Phi \left(x,y\right)=\frac{2\pi t}{{\lambda }_{0}}{n}_{g}\left(x,y\right)$$

where ${\lambda }_{0}$ is the wavelength of the incident light and *t* is the LC cell thickness.

The transmittance function of the sinusoidal phase grating is given by,

5 $${t}_{\mathrm{SP}}\left(x ,y\right)=\mathrm{exp}\left\{i\left[A+B\mathrm{cos}\left(Gx-p\theta \right)\right]\right\}$$

Using the Identity

6 $${e}^{i(\frac{m}{2})\mathrm{sin}(2\pi \vartheta x)}=\sum_{q=-\infty }^{+\infty }{J}_{q}\left(\frac{m}{2}\right){e}^{i(2\pi q\vartheta x)},$$

the transmittance function of a sinusoidal phase diffraction grating in cylindrical coordinates is expressed as follows^29^:

7 $${\mathrm{T}}_{\mathrm{P}}(\mathrm{r},\uptheta )=\sum_{\mathrm{m}=-\infty }^{\infty }{\mathrm{t}}_{\mathrm{m}}\mathrm{exp}\{-\mathrm{im}(\mathrm{Grcos\varphi }-\mathrm{p\theta })\}$$

where $\left(\mathrm{r},\mathrm{\varphi }\right)$ are polar coordinates of the grating plane, p is an integer that shows the nonlocality of fork grating and is called the defect number of the grating. When p = 0, the fork grating converts to blaze grating. The features of transmittance coefficient ${\mathrm{t}}_{\mathrm{m}}$ depend on the grating type.

In this work, a binary amplitude fork grating with *p* = 1, has been used as a mask in front of the LC cell which produces a quasi-sinusoidal phase (and not amplitude) grating in the cell.

### Diffraction of a Gaussian beam from a fork-shaped structure: general formulation

Considering $(x,y,z)$ as the coordinates in the Cartesian coordinates and $(r,\theta ,z)$ as the corresponding cylindrical coordinates, the generalized transmission function of a fork-shaped structure can be expressed as follows:

8 $$T\left(x,y\right)=\mathrm{Pe}\left(\frac{2\pi }{d}x-p\theta \right),$$

where $\mathrm{Pe}(u)$ is an arbitrary periodic function with fundamental period of $2\pi$ and $d$ is spatial period of the grating far from the singularity point, and $p$ is an integer. Considering Fourier expansion of $\mathrm{Pe}(u)$, Eq. (8) can be rewritten as follows^11^:

9 $$T\left(x,y\right)=\mathrm{ Pe}(u)=\sum_{m=-\infty }^{+\infty }{t}_{m}\mathrm{exp}\left(imu\right),$$

in which ${t}_{m}$ indicates Fourier coefficients and $u=\left(\frac{2\pi }{d}x-p\theta \right)$. In the diffraction of a light beam from this structure the power contribution of the $m$ th diffraction order can be obtained by calculating $\frac{{\left|{t}_{m}\right|}^{2}}{\sum_{n=-\infty }^{+\infty }{\left|{t}_{n}\right|}^{2}}$ According to Parseval’s relation

10 $$\sum_{n=-\infty }^{+\infty }{\left|{t}_{n}\right|}^{2}=\frac{1}{2\pi }{\int }_{-\pi }^{\pi }{\left|\mathrm{Pe}(u)\right|}^{2}du,$$

where for the special case in which $T\left(x,y\right)$ is a pure phase structure, say $\mathrm{Pe}\left(u\right)=\mathrm{exp}\left[if(u)\right]$, Eq. (10) leads to $\sum_{n=-\infty }^{+\infty }{\left|{t}_{n}\right|}^{2}=1 ,$ and power contribution of the $m$ th diffraction order is equal to ${\left|{t}_{m}\right|}^{2}$.

Now assume that a fork-shaped structure is illuminated by a Gaussian beam so that the waist of the beam is on the $z=0$, namely the aperture plane. There, for the complex amplitude immediately after the structure is

11 $$\psi \left(x,y,0\right)=T\left(x,y\right)\mathrm{exp}\left(-\frac{{x}^{2}+{y}^{2}}{{w}_{0}^{2}}\right),$$

where ${w}_{0}$ indicates the waist radius of the incident Gaussian beam. The complex amplitude of the light at distance $z$ from the structure is predictable by Fresnel integral, expressed as the following form^30^:

12 $$\psi \left(x,y,z\right)=h{\int }_{-\infty }^{+\infty }{\int }_{-\infty }^{+\infty }{e}^{i\alpha \left[{x {^{\prime}}}^{2}+{y{^{\prime}}}^{2}-2(x{x}^{\mathrm{^{\prime}}}+yy{^{\prime}})\right]}\psi \left(x{^{\prime}},y{^{\prime}},0\right)d{x}^{{{\prime}}}d{y}^{{{\prime}}},$$

in which $\alpha =\frac{\pi }{\lambda z}$ and

13 $$h=h\left(x,y,z\right)=\frac{\mathrm{exp}\left[ikz+i\alpha \left({x}^{2}+{y}^{2}\right)\right]}{i\lambda z},$$

where $k=\frac{2\pi }{\lambda }$ is the wavenumber. By substituting Eq. (9) in Eq. (11) and replacing the result in Eq. (12) we get

14 $$\psi \left(x,y,z\right)=h\sum_{m=-\infty }^{+\infty }{t}_{m}{\int }_{-\infty }^{+\infty }{\int }_{-\infty }^{+\infty }{e}^{-\frac{{x{^{\prime}}}^{2}+{y{^{\prime}}}^{2}}{{w}^{2}}-2i\alpha \left({X}_{m}{x}^{{{\prime}}}+y{y}^{{{\prime}}}\right)}{e}^{-ipm{\theta }^{{{\prime}}}}d{x}^{{{\prime}}}d{y}^{{{\prime}}},$$

where $\frac{1}{{w}^{2}}=\frac{1}{{w}_{0}^{2}}-i\alpha$ and ${X}_{m}=x-m\frac{\lambda z}{d}$ . It is noteworthy that $m\frac{\lambda z}{d}$ denoted the coordinates of the $m$ th diffraction order at plane $z$^31^. Now let us define following parameters as the polar coordinates around the center of $m$ th diffraction order as follows: ${\rho }_{m}=\sqrt{{X}_{m}^{2}+{y}^{2}}$ and ${\varphi }_{m}={\mathrm{tan}}^{-1}\left(\frac{y}{{X}_{m}}\right)$ and therefore we can write ${X}_{m}={\rho }_{m}\mathrm{cos}{\varphi }_{m}$ and $y={\rho }_{m}\mathrm{sin}{\varphi }_{m}$ . Then using substituting these parameters Eq. (13) can be rewritten in the polar coordinates:

15 $$\psi \left(x,y,z\right)=h\sum_{m=-\infty }^{+\infty }{t}_{m}{\int }_{0}^{2\pi }{\int }_{0}^{\infty }{e}^{-{\left(\frac{{r}^{{{\prime}}}}{w}\right)}^{2}-2i\alpha {\rho }_{m}{r}^{{{\prime}}}\mathrm{cos}\left({\theta }^{{{\prime}}}-{\varphi }_{m}\right)}{e}^{-ipm{\theta }^{{{\prime}}}}{r}^{{{\prime}}}dr{^{\prime}}d{\theta }^{{{\prime}}},$$

where we used ${x}^{{{\prime}}}=r{^{\prime}}\mathrm{cos}{\theta }^{{{\prime}}}$ and ${x}^{{{\prime}}}=r{{\prime}}\mathrm{sin}{\theta }^{{{\prime}}}$ in which $({r}^{{{\prime}}},{\theta }^{{{\prime}}})$ denotes the polar coordinates in observation plane. Now using the following expansion^32^:

16 $${e}^{-2i\alpha {\rho }_{m}{r}^{{{\prime}}}\mathrm{cos}\left({\theta }^{{{\prime}}}-{\varphi }_{m}\right)}=\sum_{n=-\infty }^{+\infty }{i}^{-\left|n\right|}{J}_{\left|n\right|}\left(2\alpha {\rho }_{m}{r}^{{{\prime}}}\right){e}^{-in\left({\theta }^{{{\prime}}}-{\varphi }_{m}\right)},$$

Equation (14) can be rewritten as follows:

17 $$\psi \left(x,y,z\right)=h\sum_{m,n=-\infty }^{+\infty }{t}_{m}{i}^{-\left|n\right|}{e}^{in{\varphi }_{m}}{\int }_{0}^{2\pi }{e}^{-i(n+mp){\theta }^{{{\prime}}}}d{\theta }^{{{\prime}}}{\int }_{0}^{\infty }{J}_{\left|n\right|}\left(2\alpha {\rho }_{m}{r}^{{{\prime}}}\right){e}^{-{\left(\frac{{r}^{{{\prime}}}}{w}\right)}^{2}}{r}^{{{\prime}}}d{r}^{{{\prime}}}.$$

By considering $\frac{1}{2\pi }{\int }_{0}^{2\pi }{e}^{-i(n+mp){\theta }^{{{\prime}}}}d{\theta }^{{{\prime}}}={\delta }_{n,\left(-mp\right)}$ in which ${\delta }_{a,b}$ indicates Kronecker delta, Eq. (16) reduces to

18 $$\psi \left(x,y,z\right)=h\sum_{m=-\infty }^{+\infty }{t}_{m}{i}^{-\left|mp\right|}{e}^{-imp{\varphi }_{m}}{\int }_{0}^{\infty }{e}^{-{\left(\frac{{r}^{{{\prime}}}}{w}\right)}^{2}}{J}_{\left|ml\right|}\left(2\alpha {\rho }_{m}{r}^{{{\prime}}}\right){r}^{{{\prime}}}d{r}^{{{\prime}}}.$$

Using the following integral^33^:

19 $${\int }_{0}^{\infty }{e}^{-a{r}^{2}}{J}_{\nu }\left(br\right)rdr=\frac{b\sqrt{\pi }}{8{a}^{3/2}}{e}^{-\frac{{b}^{2}}{8a}}\left[{I}_{\frac{\nu -1}{2}}\left(\frac{{b}^{2}}{8a}\right)-{I}_{\frac{\nu +1}{2}}\left(\frac{{b}^{2}}{8a}\right)\right],$$

in which ${I}_{\frac{\nu \pm 1}{2}}\left(x\right)$ indicates modified Bessel functions of order $\frac{\nu \pm 1}{2}$ , Eq. (17) reduces to

20 $$\psi \left(x,y,z\right)=h{w}^{2}\sum_{m=-\infty }^{+\infty }{c}_{m}{e}^{-imp{\varphi }_{m}}{\mathcal{R}}_{m}{e}^{-{\mathcal{R}}_{m}^{2}}\left[{I}_{\frac{\left|mp\right|-1}{2}}\left({\mathcal{R}}_{m}^{2}\right)-{I}_{\frac{\left|mp\right|+1}{2}}\left({\mathcal{R}}_{m}^{2}\right)\right],$$

where ${\mathcal{R}}_{m}=\frac{\pi }{\sqrt{2}}\frac{w}{\lambda z}{\rho }_{m}$ is a dimensionless radial parameter and ${c}_{m}=\frac{{(2\pi )}^{3/2}}{4}{i}^{-\left|mp\right|}{t}_{m}$.

### Some practical examples

Let us now consider some practical examples.

#### Pure amplitude sinusoidal fork-shaped grating

Transmission function of a pure amplitude sinusoidal fork-shaped grating can be expressed as follows:

21 $$T\left(x,y\right)=\frac{1}{2}\left[1+\mathrm{cos}\left(\frac{2\pi }{d}x-p\theta \right)\right].$$

Comparing Eqs. (21) and (9) one can deduce that ${t}_{0}=\frac{1}{2}$ , ${t}_{\pm 1}=\frac{1}{4}$ and other coefficients vanish. Substituting these coefficients in Eq. (20) complex amplitude of the diffracted light can be calculated, see Visualization1.

#### Pure amplitude binary fork-shaped grating

Transmission function of a pure amplitude binary fork-shaped grating can be expressed as follows:

22 $$T\left(x,y\right)=\frac{1}{2}\left\{1+\mathrm{sgn}\left[\mathrm{cos}\left(u\right)\right]\right\},$$

where $u=\frac{2\pi }{d}x-p\theta$ and “$\mathrm{sgn}$” indicates sign function. The Fourier expansion of this transmittance can be expressed as follows^34^:

23 $$T\left(x,y\right)=\frac{1}{2}\sum_{m=-\infty }^{\infty }\mathrm{sinc}\left(m/2\right)\mathrm{exp}(imu).$$

Comparing Eqs. (23) and (9) one can deduce that ${t}_{m}=\frac{1}{2}\mathrm{sinc}\left(\frac{\mathrm{m}}{2}\right)$. Substituting these coefficients in Eq. (20) complex amplitude of the diffracted light can be calculated.

#### Pure phase sinusoidal fork-shaped structure

Transmission function of a pure phase sinusoidal fork-shaped grating can be expressed as follows:

24 $$T\left(x,y\right)={e}^{i\gamma \mathrm{cos}u}=\sum_{m=-\infty }^{+\infty }{i}^{m}{J}_{m}( \gamma ){e}^{imu},$$

where $\gamma$ is the phase depth of the structure. Comparing Eqs. (24) and (9) one can deduce that ${{t}_{m}=i}^{m}{J}_{m}( \gamma )$ and other coefficients vanish. Substituting these coefficients in Eq. (20) complex amplitude of the diffracted light can be calculated.

25 $$\gamma =\frac{\pi t}{{\lambda }_{0}}\Delta n.$$

In which $\Delta n$ is the refractive index anisotropy and $t$ is the LC cell thickness.

#### Pure phase binary fork-shaped structure

Transmission function of a pure phase sinusoidal fork-shaped grating can be expressed as follows:

26 $$T\left(x,y\right)={e}^{i\gamma \mathrm{sgn}\left[\mathrm{cos}\left(u\right)\right]}$$

where $\gamma$ is the phase depth of the structure. The Fourier expansion of this transmittance can be expressed as follows^35^:

27 $$T\left(x,y\right)=\mathrm{cos }\gamma +\sum_{\begin{array}{c}m=1\\ odd\end{array}}^{\infty }\frac{2{(i)}^{m}}{m\pi }\mathrm{sin}\gamma \left({e}^{imu}+{e}^{-imu}\right) ,$$

where “odd” under the summation indicates that $m$ is an odd number. Here, again Comparing Eqs. (27) and (9) one can deduce that ${t}_{0}=\mathrm{cos }\gamma$ and ${t}_{m}=\frac{2{(i)}^{m}}{m\pi }\mathrm{sin}\gamma$ for odd values of $m$ and ${t}_{m}=0$ for even values of ${t}_{m}$. Substituting these coefficients in Eq. (20) complex amplitude of the diffracted light can be calculated.

## Methods and materials

Considering LC-based electro-optical devices, these materials are widely applied in amplitude and phase modulation including LCDs, SLMs, and spiral phase plates which are made of liquid crystal cell structures^36,37^. LCs are an interesting material because of their large birefringence and electrical controllability^19^.

In this experimental work, a DDLC cell was prepared with a parallel arrangement containing E7 as the host material doped with Methyl Red dye as a guest substance. All the materials were purchased from Merc, and were used without further purification.

Table 1 shows the features of the materials used at a temperature of 25℃ and a wavelength of 632.8 nm.

**Table 1:** The characteristics of E7 Liquid crystal at a temperature of 25 °C and a wavelength of 632.8 nm^38,39^.

Chemical structure of E7	${\mathrm{n}}_{\mathrm{e}}$	${\mathrm{n}}_{\mathrm{o}}$	$\Delta \mathrm{n}$	${\mathrm{T}}_{\mathrm{NI}}\,({^\circ{\rm C} })$
	1.7305	1.5189	0.2116	58

**Figure 3:**
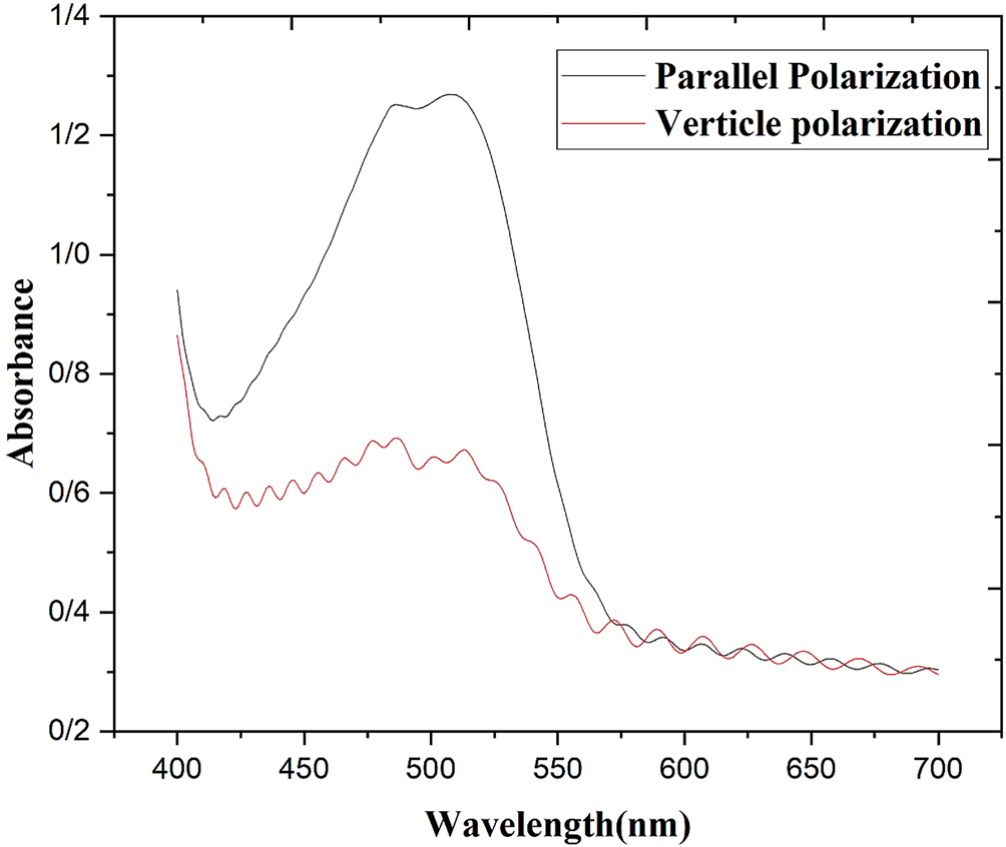
The absorption spectrum of the sample in two states, the linear incident light polarization, $\overrightarrow{\mathrm{P}}$, parallel and perpendicular to the alignment direction of the cell, $\overrightarrow{\mathrm{L}}$.

A homogeneously aligned (HG) LC cell with a thickness of 20 µm has been used as the sample. The prepared mixtures (E7 doped with MR 1 wt%) are filled by the capillary method. Each substrate contains ITO and PVA layers, and the PVA surface is grooved parallel ($\mathrm{\alpha }=0$). The absorption spectrum of the sample is shown in Fig. 3, which was obtained using a Shimadzu spectrophotometers model 2450 UV–VIS.

**Figure 4:**
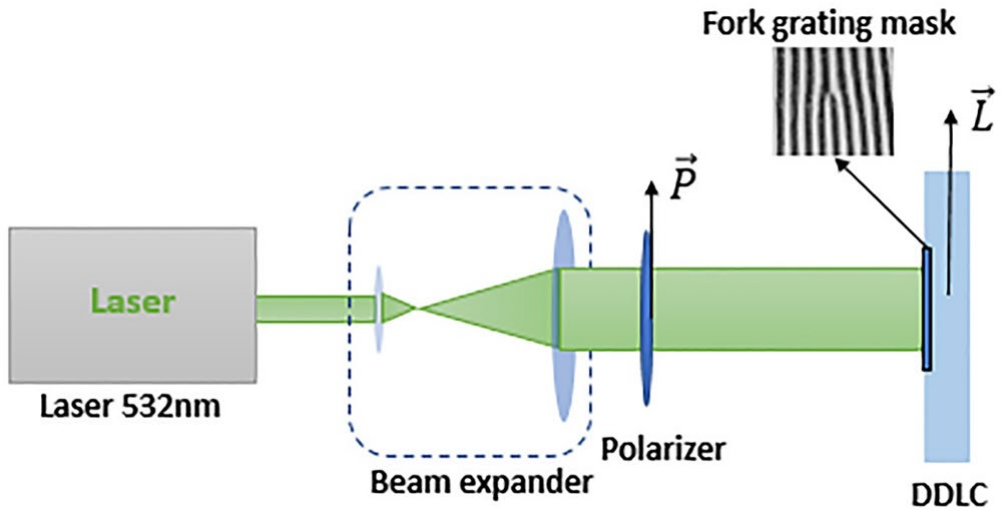
The schematic of the experimental setup to record the fork grating pattern onto cell, $\overrightarrow{\mathrm{L}}||\overrightarrow{\mathrm{P}}$.

The absorption spectrum plot of the sample shows that the wavelength of 532 nm is suitable for storing the pattern on the liquid crystal cell, thus a pump laser with a wavelength of 532 nm is selected. In addition, it is necessary to choose the polarization of the radiation to be parallel to the alignment direction of the cell that has been specified by $\overrightarrow{L}$^21^. To store the fork grating pattern on the sample, as shown in Fig. 4 first, a lithography mask is prepared based on Eq. (9) and then placed in front of the sample and illuminated by parallel linear polarized light. The mask’s dimension was 5 × 5 mm^2^ and its grating period was 0.1 mm.

**Figure 5:**
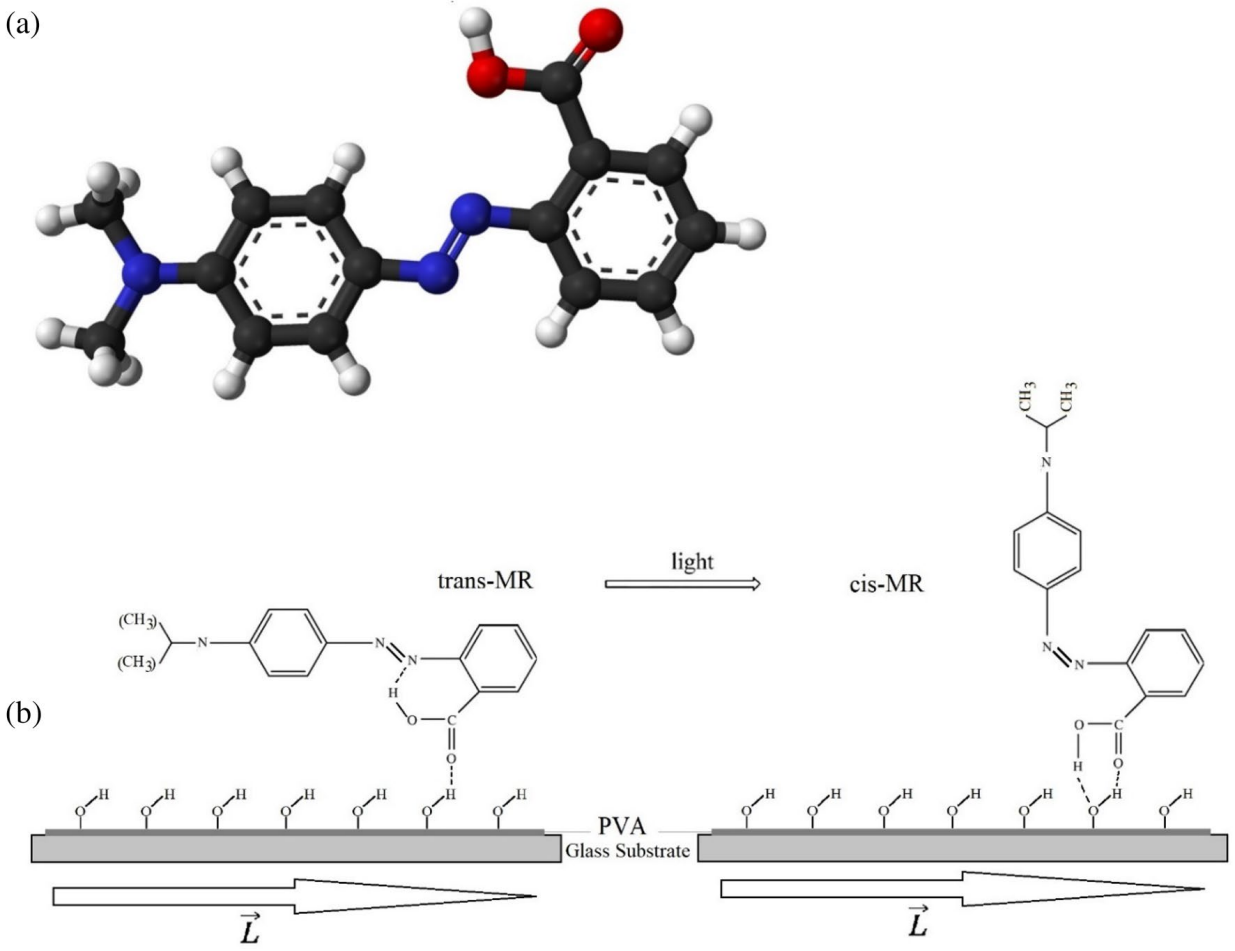
(**a**) The chemical structure of MR. (**b**) The spatial arrangement of the MR molecules on the PVA surface of the cell; before illumination, weak anchoring, and the molecules are in trans form and parallel to the alignment and after illumination, they change to cis form and due to hard anchoring the molecules are perpendicular to the alignment.

The direction of the microgrooves in the cell ($\overrightarrow{\mathrm{L}}$) determines the anisotropy of the surface, which due to anchoring energy between LC molecules and molecules of the substrate surface cause the orientation of the molecules to be parallel to the direction of the grooves^40^. By illuminating the sample using the pump beam, the process of photoisomerization of methyl red commences. Based on the molecular structure of MR azo molecules, its cis isomer in compression to trans one interacts by harder anchoring energy upon the polymer surface of the cell (PVA)^41^. According to the mask pattern, in some parts of the sample that are illuminated by the pump beam, the anchoring of cis isomers causes the MR molecules and consequently LC molecules to orient perpendicular to the cell surface. Nevertheless, in the parts that have not been illuminated by the incident beam, all of the dye molecules, following the original arrangement of the cell, remain parallel to the direction of the cell microgrooves. The process is shown in Fig. 5.

**Figure 6:**
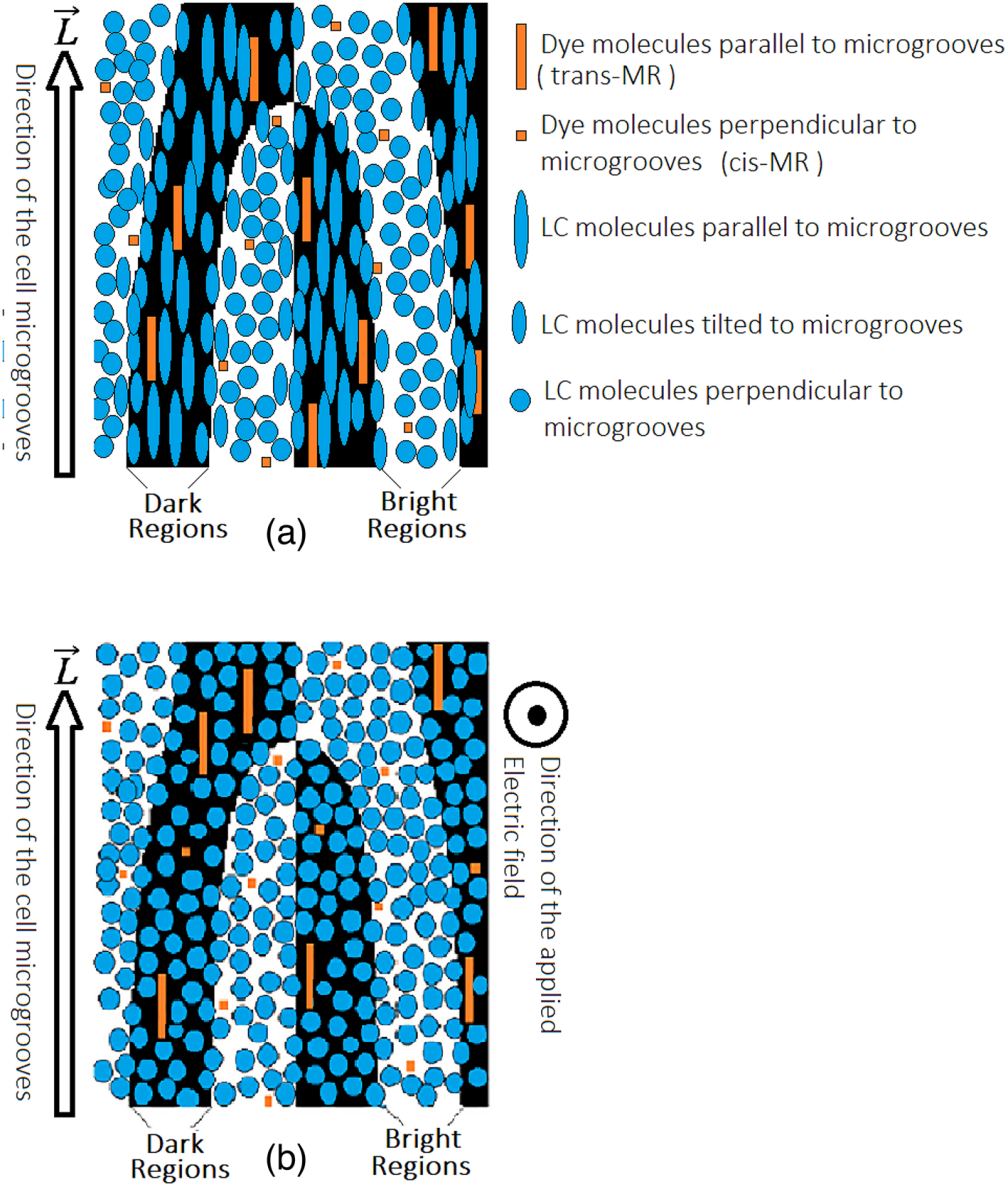
Top view for the planar cell: the rubbing angle is $\alpha =0^\circ$. (**a**) LC director distribution at 0 V, in the border of the dark and the bright regions the orientation of the LC molecules due to their associated behavior continuously and gradually passes from the parallel to perpendicular state, which in turn causes a quasi-sinusoidal phase state. (**b**) By applying a proper voltage to the sample, the director of all the LC molecules reorients and remains perpendicular to the cell surface but due to the low dipole moment of dye molecules they do not reorient under 22-V applied voltages except a little part.

It is significant to note that in the boundary of light and dark regions, the orientation due to the associated behavior of LC molecules, continuously and gradually, passes from the parallel to the perpendicular state, which in turn causes a quasi-sinusoidal phase state at the edges. So, as mentioned in the theory section, according to the formation of such a quasi-sinusoidal phase grating in the sample, it is expected that this grating will be able to convert the Gaussian incident beam to the vortex beam. Figure 6a explains this process. After applying an electric field to the sample, the molecules reorient and are placed perpendicular to the cell surface, as illustrated in Fig. 6b. It leads to eliminating the generated quasi-sinusoidal phase grating and inside the cell a homogenous and continuous region is created which is expected to destroy the diffraction effect completely.

**Figure 7:**
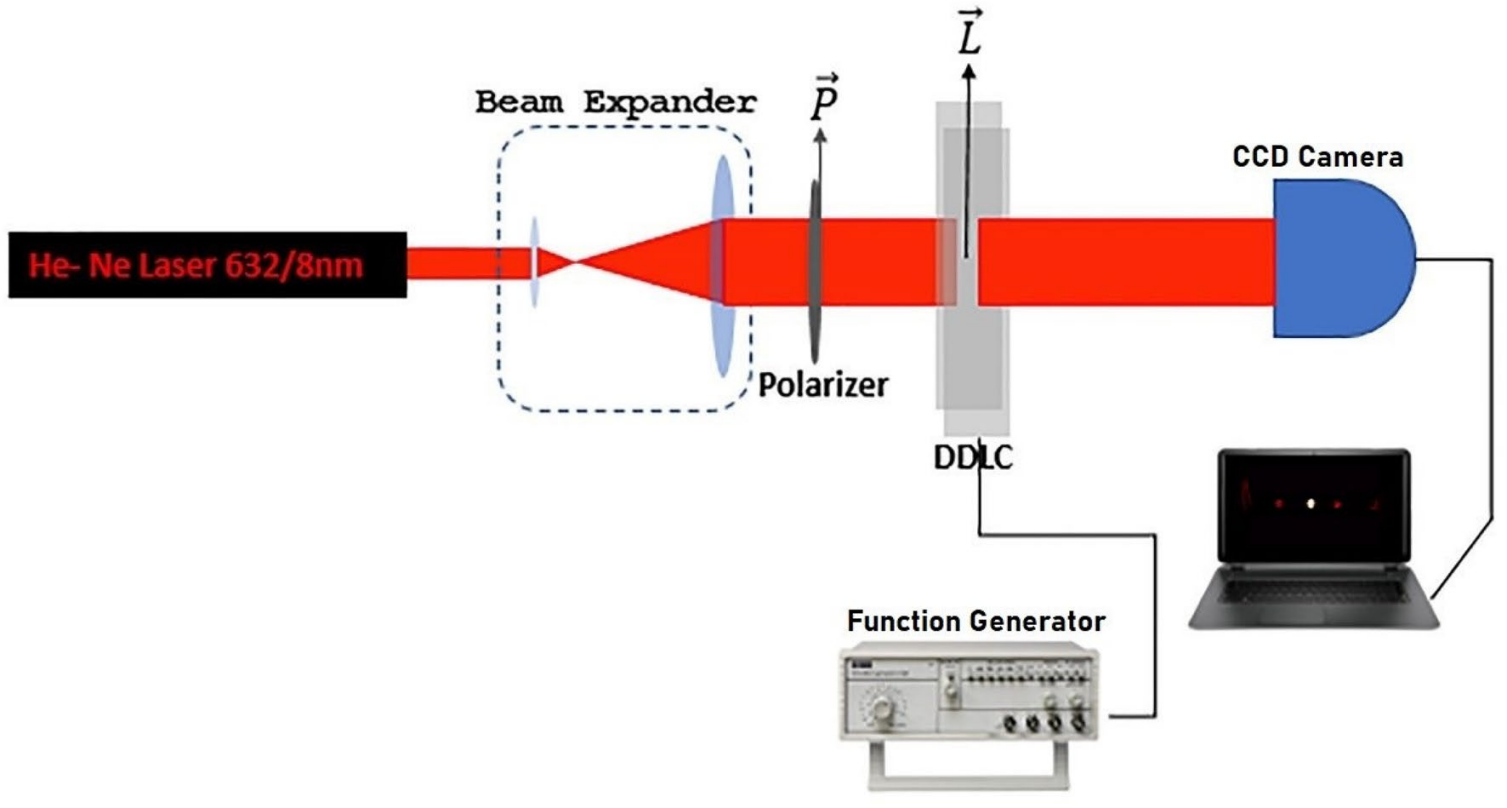
The schematic of vortex beam generation by using DDLC cell.

After the grating pattern was stored, the He–Ne laser with linear polarization was used as a probe beam to produce a vortex beam via the sample. The light absorption of the probe beam at a wavelength of 632.8 nm is diminutive, therefore the studied grating is accepted as a type of phase grating which is shown in Fig. 7. The incident light polarization is considered parallel to the alignment of the cell ($\overrightarrow{\mathrm{L}}$), and a CCD camera is used to record the diffraction pattern. A function generator is used to apply a square wave AC voltage with a 500 Hz frequency to the cell.

## Result and discussion

**Figure 8:**
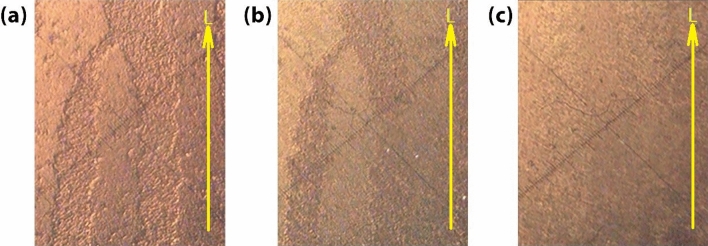
The surface morphology of the stored fork grating (singularity topological charge p = 1) in the sample is imaged using a polarizing microscope under (**a**) 0 v, (**b**) 3.9 v, and (**c**) 22 v applied voltage.

The analysis of surface morphology of the singularity structure of the sample in different applied voltages was recorded using a polarizing microscope and is shown in Fig. 8. The recorded topological charge for the stored fork grating is p = 1. According to the results, without applying voltage, it is observed that there was a refractive index anisotropy between the illuminated and non-illuminated areas inside the cell. In these situations, there is a transverse distribution of anisotropy and heterogeneity in the orientation of liquid crystal molecules on the cell surface. When the voltage is not applied, the dark areas of the sample for the passing probe beam have a refractive index of ${n}_{o}$ and the light areas have a refractive index of ${n}_{e}$(q)*,* which are the ordinary and extraordinary refractive indices of the liquid crystal, respectively. Accordingly, the main difference between the pattern stored areas versus the not stored areas is in the orientation style of the molecules and the refractive index felt by the incident beam. In Fig. 8b, the phase difference in the recording areas has been reduced slightly as a result of the relative voltage increase up to 3.9 v. The anisotropy of the sample is entirely removed by increasing the voltage up to 22 v, resulting in a homogeneous medium in the cell, as shown in Fig. 8c. In this case, it is expected that the light encounters only one refractive index, so the phase difference disappears. By eliminating the phase contrast between the light and dark regions of the stored fork grating, the diffraction beams and as a result, the produced vortex beams will be eliminated.

**Figure 9:**
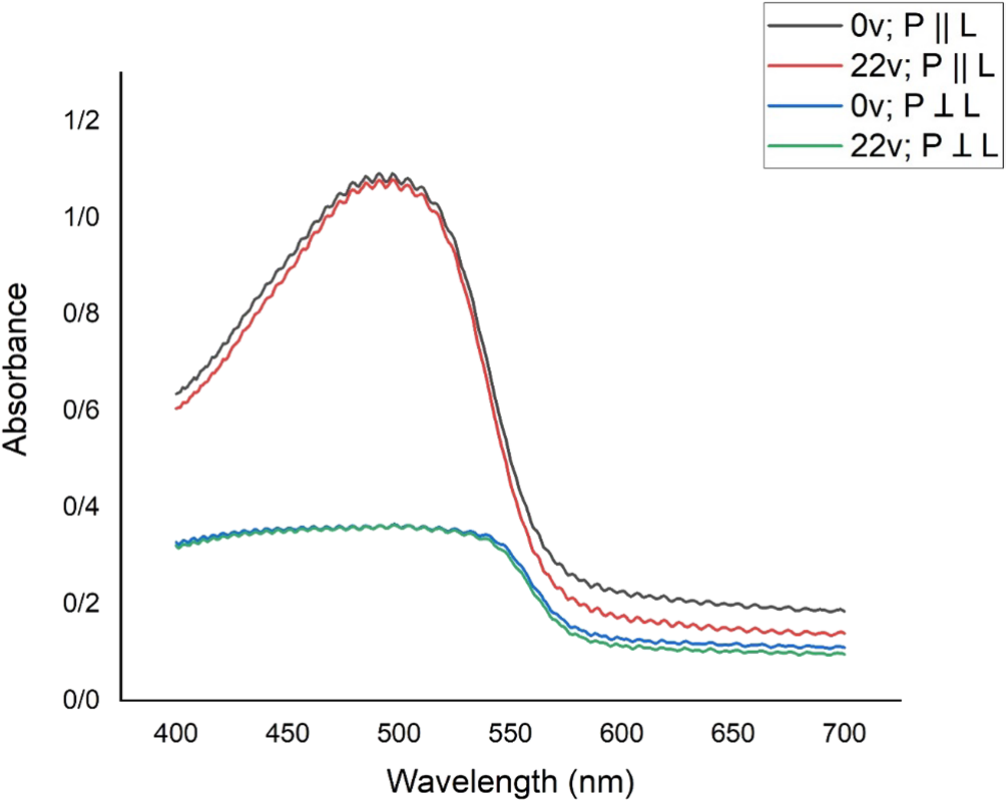
The plot of absorption spectrum in parallel and perpendicular polarization proportion to the alignment of sample at different applied voltages. Results show that as predicted in Fig. 6, applied voltage do not affect dye molecules’ reorientation.

To investigate the effect of the applied voltage on the absorption spectrum of the sample, they were studied under different voltages after the pattern was stored. After the investigation, it was obvious that the applied voltage did not affect the absorption spectrum of the sample at the probe wavelength of 632.8 nm, as shown in Fig. 9.

The results in Fig. 9 show that applying low voltages which are able to reorient the LC molecules unable to reorient dye molecules and it seems it could be due to their low dipole moment with respect to the LCs.

**Figure 10:**
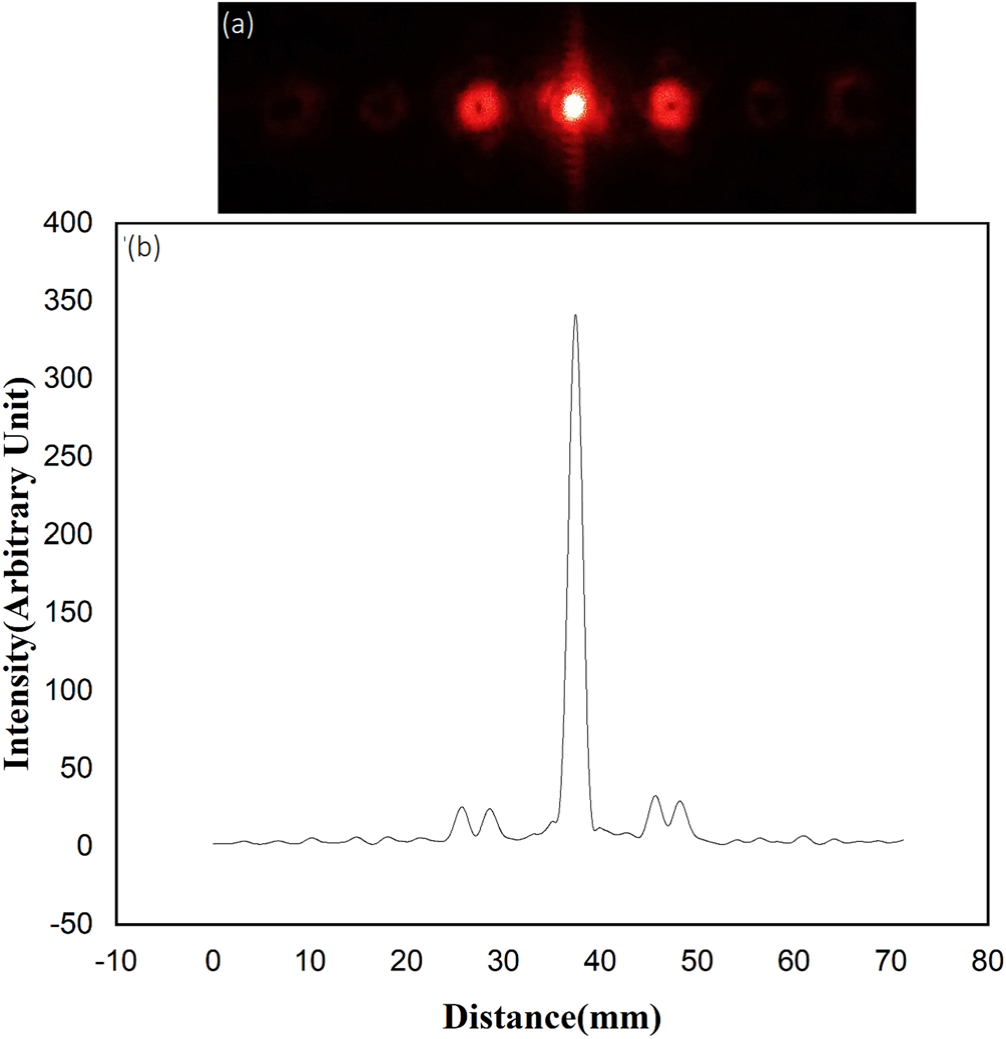
(**a**) The image of the diffracted beam from the stored pattern in the sample without applying voltage, (**b**) the profile of the diffracted beam.

In Fig. 10, the image of the diffracted beam from the stored pattern in the sample without applying voltage is shown. As shown in Fig. 10a, up to the 3rd order of diffraction can be presented. As it was discussed in the theory, a defect in the grating causes a singularity in the produced vortex beam. This singularity can be seen in the center of the diffracted vortex beams as a dark spot center and a bright ring around it. The vortex beam generated by the phase grating, using the applied voltage is switched in a controlled manner. Figure 10b shows the transverse intensity distribution of different diffraction orders. The profile of the first order diffraction is similar to the distribution function of the Laguerre Gaussian beam, which indicates the production of vortex beams.

**Figure 11:**
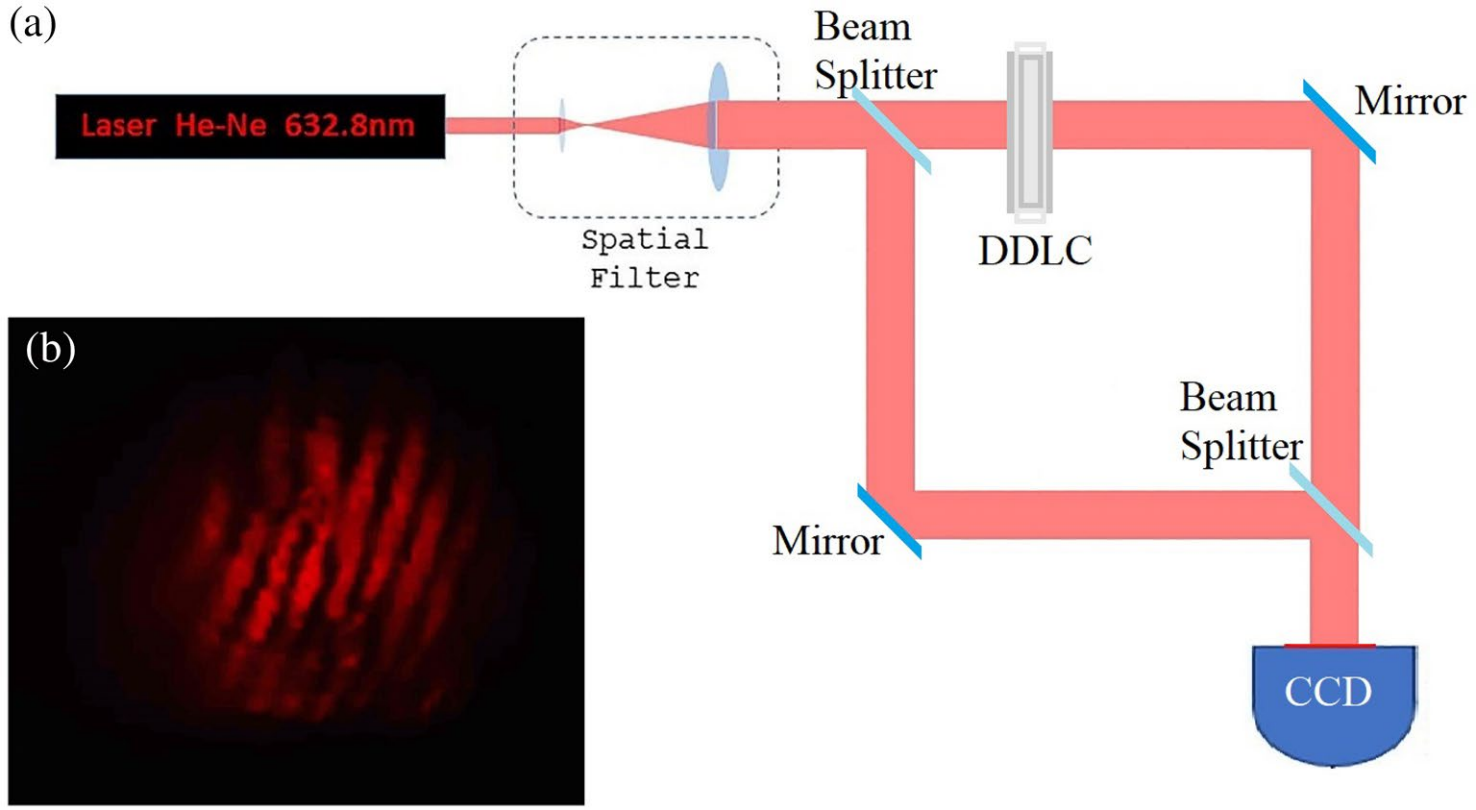
(**a**) The interference set-up to detection of topological charge of created vortex beam, (**b**) interference pattern shows the Fork grating with p = 1.

### Detection of topological charge

To detect the topological charge of the created vortex beam, an interference method was used. According to the setup shown in Fig. 11 by interfering with the incident beam by one of the diffracted vortex beams, the Fork structure appears that shows the topological charge of a created vortex beam is *p* = 1.

**Figure 12:**
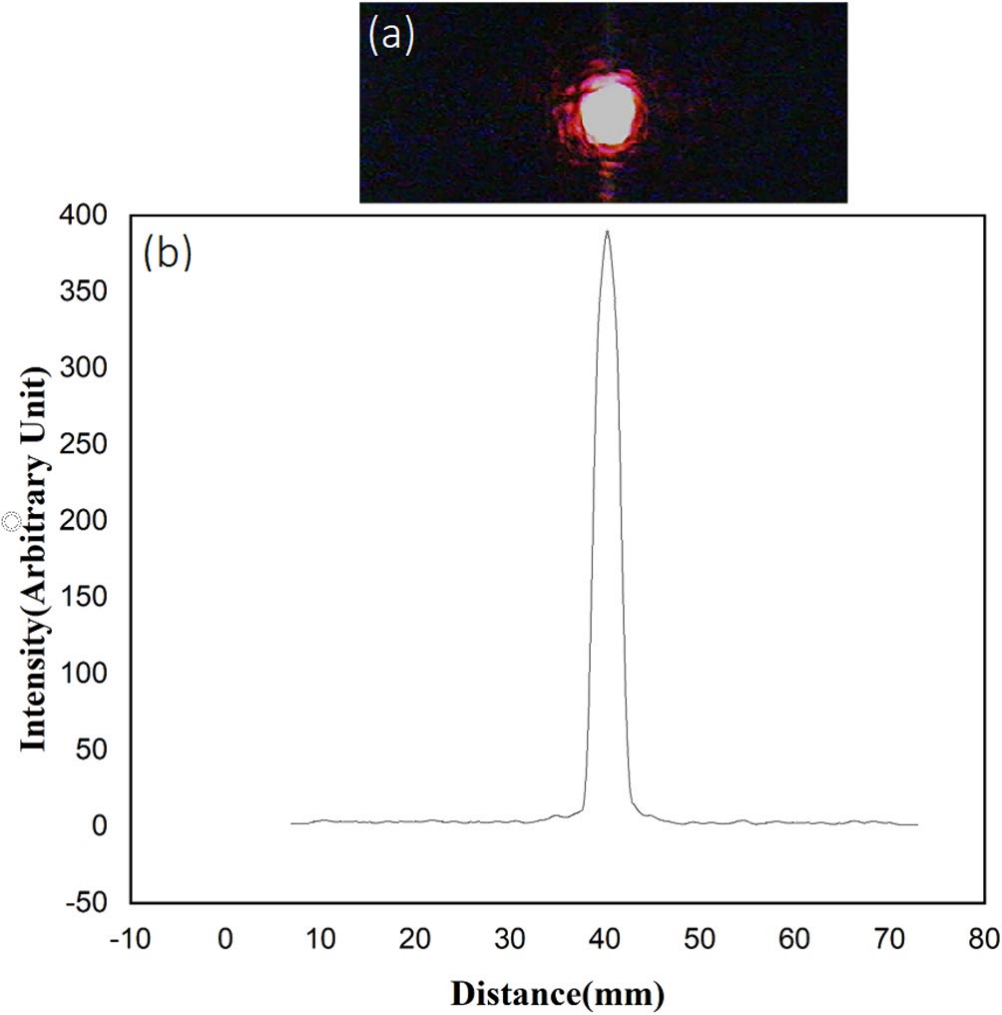
(**a**) The beam passed through the sample after applying a voltage of 22 v, and (**b**) the transmitted beam profile.

By increasing the voltage, as expected and explained in molecular orientation analysis in Fig. 6, all the diffraction orders are removed and only the central beam remains. The image of the passing beam and its profile after applying 22 Volts voltage is shown in Fig. 12.

**Figure 13:**
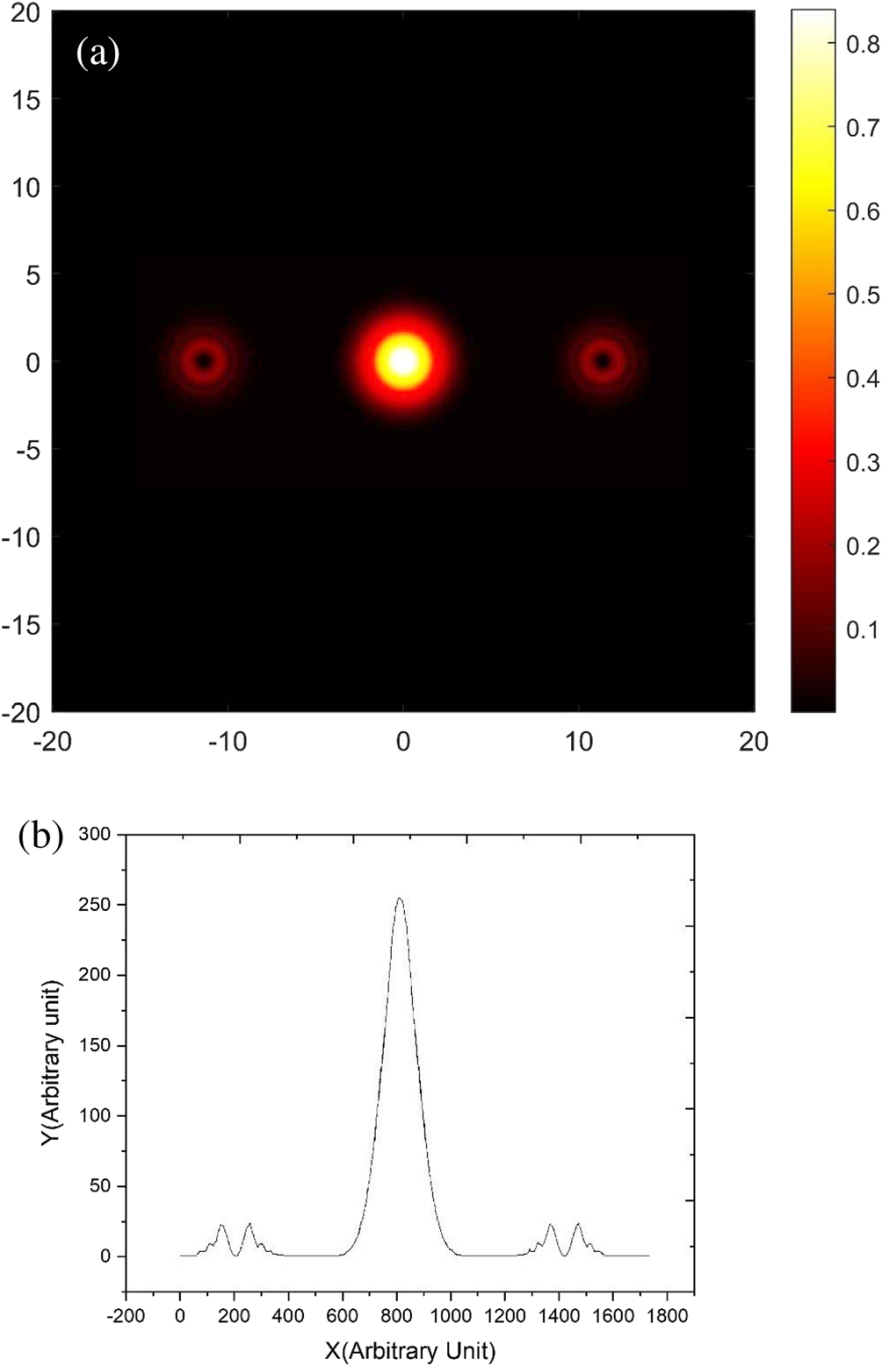
(**a**) The simulated image of the diffracted beam from the fork shape phase grating, (**b**) the corresponding intensity profile of the grating with 8% diffraction efficiency.

Results obtained from MATLAB simulations related to the theoretical aspects introduced in pure phase sinusoidal fork-shaped structure section shows that, as it has obtained experimentally for quasi-sinusoidal phase grating, the diffracted beam has the optical vortex beam properties with topological charge *p* = 1. Figure 13 shows the simulation results based on the equations introduced in theory section.

**Figure 14:**
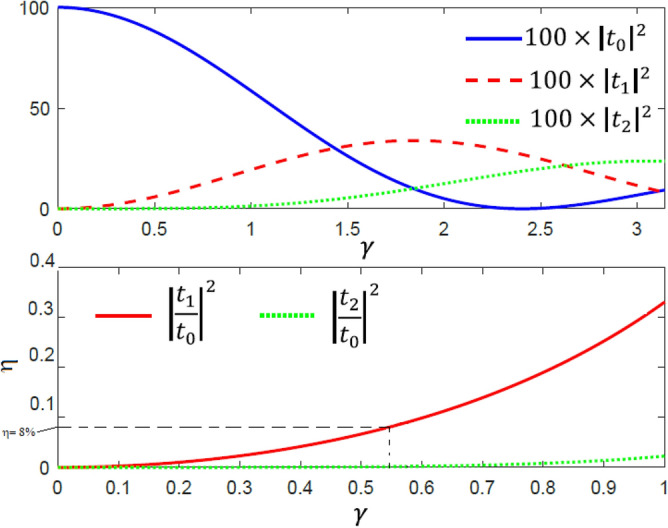
The transmittance and diffracted efficiency diagrams obtained by simulation results for the LC cell with the cell thickness t = 20 micron and incident wavelength ${\uplambda }_{0}=632.8\mathrm{ nm}.$

The simulation results enable us to calculating the refractive index anisotropy created in the cell between dark and bright regions. According to the experimentally obtained diffraction efficiency ($\eta =8\%$) and comparing it with diffraction efficiency diagram obtained by simulation, Fig. 14, and using Eq. (25) it is obvious that the refractive index anisotropy in the cell is $\Delta n={n}_{e}\left(\theta \right)-{n}_{o}=5.8\times {10}^{-3}.$ According to Table 1 and using the refractive index ellipsoid equation, the simulation data for the sample indicates that the Cis form of MR molecules in bright regions has been able to change the angle of LC molecules on average about 12.7° from parallel toward a vertical position with respect to the $\overrightarrow{L}$. This orientation angle would be depended on the dye concentration which was 1 wt%.

**Figure 15:**
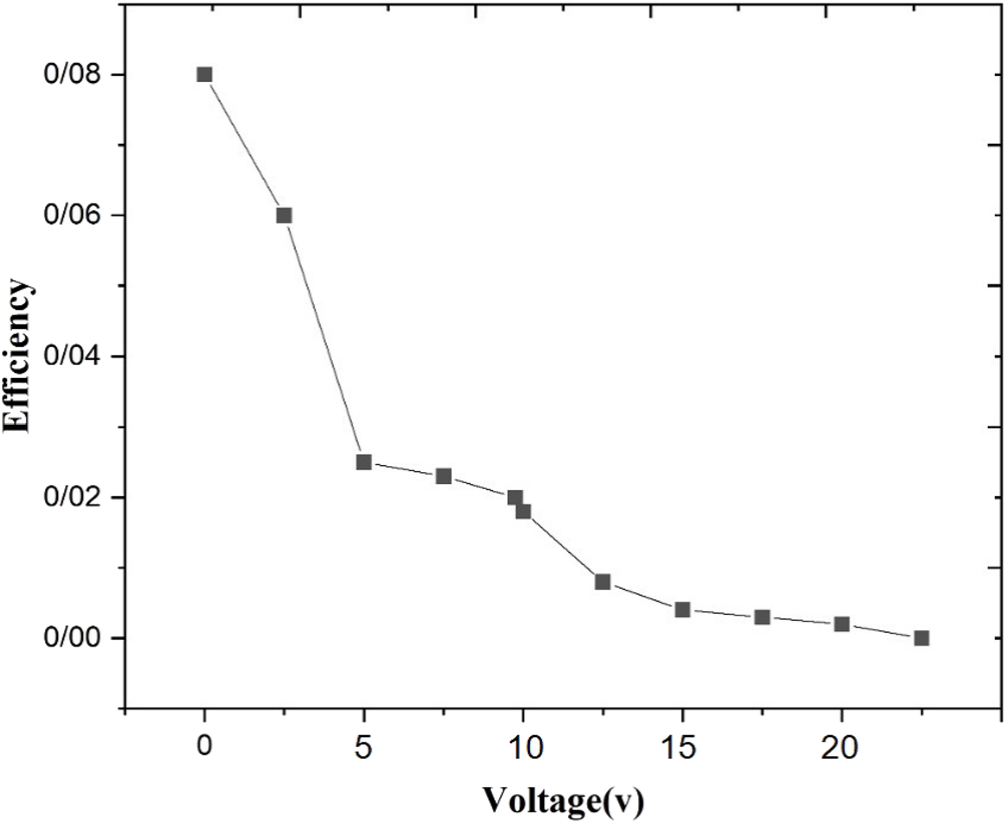
The plot of first order diffraction efficiency versus the applied voltage.

Experimentally obtained results as shown in Fig. 15 indicates that the first order diffraction efficiency how decreases with the increase of voltage, as it explained in theory section.

**Figure 16:**
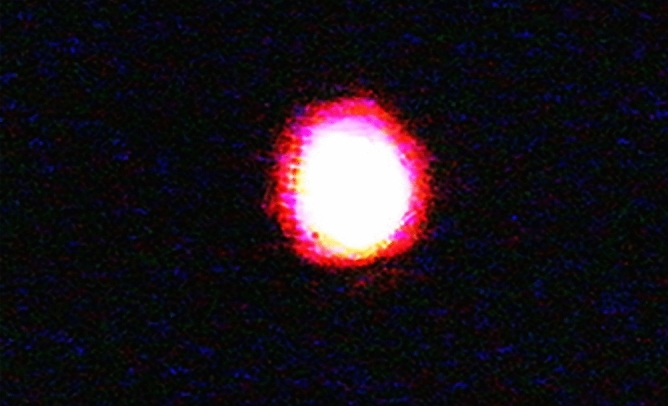
The image of a transmitted probe beam through the sample (its polarization is perpendicular to the cell alignment) at 0 v applied voltage.

Furthermore, in the second part of the results, an incident beam with perpendicular polarization ($\overrightarrow{L} \perp \overrightarrow{P}$) was also applied. The result is shown in Fig. 16. The output beam has a Gaussian intensity distribution, and there is no diffraction beam in absence of the applied voltage.

## Conclusion

The E7 liquid crystal was doped with MR dye at 1 wt% and investigated to be controllable of generated vortex beams in presence of applied voltage. The DDLC cell was illuminated using the pump laser with the wavelength of 532 nm and a quasi-sinusoidal phase fork grating pattern was recorded on the sample due to the hard anchoring of the dye's cis-isomers on the cell surface. The stored grating on the DDLC cell shows that the liquid crystal molecules in the cell based on the light-driven reorientation of the dye molecules are oriented in different directions. The probe laser beam (He–Ne 633 nm), encounters the phase grating refractive index and due to the presence of defects in the grating structure, produces vortex beams with a Lager-Gaussian profile. By applying voltage, liquid crystal molecules with an extraordinary refractive index, reorient and change the phase contrast inside the liquid crystal cell. As the voltage continues to increase, the sample reaches an isotropic orientation distribution, so the produced diffraction order is also removed. In fact, a quasi-sinusoidal phase fork grating is produced, which is controlled via voltage. this device can be used as beam modulators, imaging systems, controllable diffraction optics, optical connectors, quantum information transmission, optical tweezers, and particle trapping.

## Data Availability

The datasets generated during and/or analysed during the current study are available from the corresponding author on reasonable request.
